# Assessment of *fib* Bulletin 90 Design Provisions for Intermediate Crack Debonding in Flexural Concrete Elements Strengthened with Externally Bonded FRP

**DOI:** 10.3390/polym15030769

**Published:** 2023-02-02

**Authors:** Alba Codina, Cristina Barris, Younes Jahani, Marta Baena, Lluís Torres

**Affiliations:** AMADE, Polytechnic School, University of Girona, 17003 Girona, Spain

**Keywords:** reinforced concrete, FRP, EBR, intermediate crack debonding

## Abstract

With the assessment of intermediate crack debonding (ICD) being a subject of main importance in the design of reinforced concrete (RC) beams strengthened in flexure with externally bonded fibre-reinforced polymer (FRP), several approaches to predict the debonding loads have been developed in recent decades considering different models and strategies. This study presents an analysis of formulations with different levels of approximation collected in the *fib* Bulletin 90 regarding this failure mode*,* comparing the theoretical predictions with experimental results. The carried-out experiments consisted of three RC beams strengthened with carbon FRP (CFRP) tested under a four-point bending configuration with different concrete strengths and internal steel reinforcement ratios. With failure after steel yielding, higher concrete strength, as well as a higher reinforcement ratio, lead to a higher bending capacity. In addition, the performance of the models is assessed through the experimental-to-predicted failure load ratios from an experimental database of 65 RC beams strengthened with CFRP gathered from the literature. The results of the comparative study show that the intermediate crack debonding failure mode is well predicted by all models with a mean experimental-to-predicted failure load ratio between 0.96 and 1.10 in beams tested under three- or four-point bending configurations.

## 1. Introduction

The strengthening of reinforced concrete (RC) structures with fibre-reinforced polymer (FRP) materials has been extensively performed in construction during the last two decades due to the advantages in the mechanical and durability properties of these materials over traditional techniques such as reinforcement with steel plates. FRPs are composite materials made of a polymeric matrix (resin) reinforced with continuous fibres of glass (GFRP), carbon (CFRP), basalt (BFRP), or aramid (AFRP). Some of the reasons why these materials are increasingly used are their durability and resistance to corrosion, high tensile strength- and stiffness-to-weight ratio, low weight resulting in ease of installation and reduction in labour costs, and large availability of sizes and geometries [1,2,3]. CFRP strengthening has been shown to increase the stiffness and strength performance of flexural members. It is currently a widespread strengthening methodology for RC members with a variety of applications, for example, the strengthening of sea sand RC members [4].

Two techniques are typically used to retrofit the RC structures with FRP materials: externally bonded reinforcement (EBR) and near-surface mounted (NSM). The EBR method is widely used to effectively strengthen RC structures in flexure, shear, and torsion, as well as to introduce favourable confinement effects [5]. However, members with flexural reinforcement often suffer from premature debonding of the FRP from the concrete surface before the sectional failure due to FRP rupture or concrete crushing [1,2,6], leading to a high underutilisation of the FRP reinforcement mechanical properties. The utilisation rate of the tensile strength may be improved by prestressing the CFRP plates before bonding them to the substrate. In the latter, the choice of the end anchorage system will be of importance to avoid a significant loss of the prestressing forces [7,8].

In the system FRP/adhesive/concrete, debonding may take place within the concrete (cohesive failure), the adhesive (cohesive failure), the laminate (delamination failure), or in the interfaces between these materials (adhesion failure). If a proper application of the strengthening system is carried out, the weakest part of the system is the concrete layer near the interface with the adhesive as the tensile strength of the concrete is usually much lower than the adhesive strength [9]. Considering the origin of the debonding, failure modes can be classified as intermediate crack debonding (ICD), starting at an intermediate section of the beam due to flexural (or flexural-shear) cracks and propagating to the support, and end debonding (ED), which occurs at the curtailment region of the FRP reinforcement. As observed from experiments in the literature, ICD is usually the governing failure mode in flexural applications [9,10].

**Figure 1:**
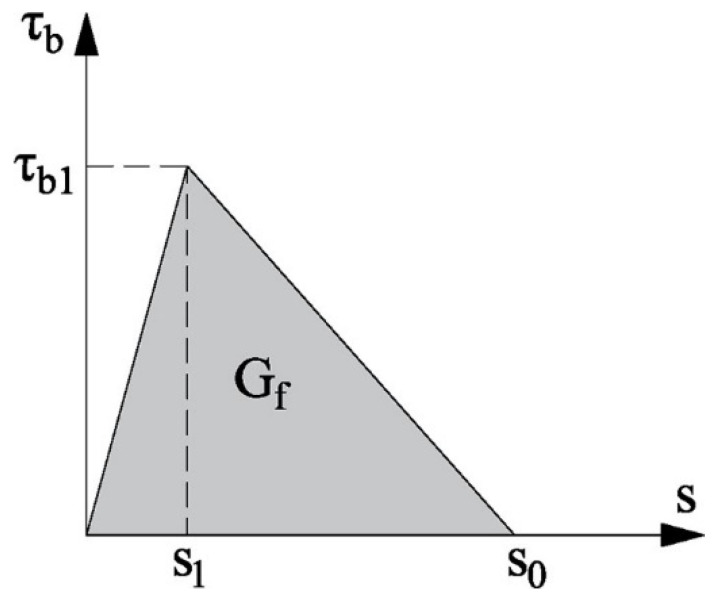
Bilinear local bond-slip law.

In an EBR FRP-concrete joint, normal stresses in the FRP are transferred to the concrete through shear stresses applied to its surface. When these stresses attain the value of the bond strength, the debonding process initiates. The bond behaviour of the interface can be described in terms of the shear stresses (*τ_b_*) and the slip (*s*) of the laminate from the substrate. Several models can be found in the literature coming from experimental assessment and simplifications [11,12,13,14,15,16,17,18]. The bond behaviour is often well represented by a bilinear bond law (Figure 1, Equation (1)) with an initial ascending branch up to the maximum shear stress *τ_b_*_1_ (bond strength), followed by a linear descending branch (due to the damage of materials) until the maximum slip *s*_0_. The fracture energy *G_f_* of the system is defined as the area under the bond stress-slip curve, which, for a bilinear law, can be expressed by Equation (2).

(1) $$\tau_{b}\left(s\right)=\left\{\begin{array}{cc} \frac{\tau_{b1}}{s_{1}}s & for\;s\leq s_{1}\\ \tau_{b1}\frac{\left(s_{0}-s\right)}{\left(s_{0}-s_{1}\right)} & for\;s>s_{1} \end{array}\right.$$

(2) $$G_{f}=\frac{\tau_{b1}\cdot s_{0}}{2}$$

This bilinear bond behaviour is usually described using direct shear tests [19,20]. Although in flexural applications (i.e., beams), curvature may cause peeling stresses perpendicular to the FRP surface, shifting the shear failure mode II to a mixed mode I-II, the normal component is usually neglected, and, as a simplification, debonding is treated as a pure shear (mode II) failure [9].

Several analytical models have been developed to predict ICD failure and adopted by codes of practice [1,2,6,21], involving different levels of approximation. There is still uncertainty in the precise role of the influencing parameters in the debonding phenomena, hence simplified design formulations based on the direct calibration of empirical expressions against experimental results are often presented in the literature. Usual models are based on different approaches: a limitation of the stress or strain in the FRP strengthening at the critical section of the beam [2,6,22,23,24]; a limitation of the maximum mean bond stress [25]; or a limitation of the increment of tensile force/stress in the FRP [21,26,27,28,29].

In the *fib* Bulletin 90 [1], different approaches to predicting ICD are presented, addressing different levels of approximation and parameters defining the bond-slip law, which may lead to different predictions. This paper aims to assess these proposals in order to understand their basis, highlight their differences, and compare their predictions with experimental results.

In this study, a detailed description of the different approaches is presented along with an experimental campaign that was performed to compare the theoretical predictions with experimental results. The experimental results are presented and discussed in terms of modes of failure, flexural capacity, load-deflection response, and load-strain response in the CFRP. As a further element of assessment, a database of 65 EBR CFRP RC beams that failed by ICD gathered from the literature is analysed using the different proposals. Experimental-to-predicted failure load ratios are presented, and the differences in the approaches are highlighted.

## 2. Analysis of the Current Formulations for ICD

Three main approaches are presented in the *fib* bulletin 90 [1] to predict ICD failure. All of them are based on the transfer of bond stresses in an FRP-strengthened concrete element between two cracks. However, the limiting value is established by following two different approaches, either the maximum stress or the maximum increment of tensile force in the FRP reinforcement.

The transfer of bond stresses is limited by the maximum transferrable (anchorage) stress in the FRP, which can be derived from a non-linear fracture mechanics (NLFM) analysis of direct shear tests [11,12,14,30,31,32]. Equation (3) is suggested to calculate the anchorage strength, where *E_f_* and *t_f_* are the elastic modulus and thickness of the FRP, respectively, and *β_l_* (Equations (4) and (5)) is the reducing factor for bonded lengths (*l_b_*) lower than the effective length (*l_e_*), thus taking into consideration that the full bond-slip law will not be developed, and the maximum anchorage force will not be attained. The effective length for a bilinear bond law can be calculated using Equation (5).

(3) $$f_{fb}\left(l_{b}\right)=\beta_{l}\left(l_{b}\right)\sqrt{\frac{2E_{f}\cdot G_{f}}{t_{f}}}$$

(4) $$\beta_{l}=\left\{\begin{array}{c} \frac{l_{b}}{l_{e}}\;\left(2-\frac{l_{b}}{l_{e}}\right)<1\;\;\mathrm{for}\;l_{b}\leq l_{e}\\ 1\;\;\;\;\;\;\;\;\;\;\;\;\;\;\;\;\;\;\;\;\;\;\;\;\;\;\;\;\;\mathrm{for}\;l_{b}>l_{e} \end{array}\right.$$

(5) $$l_{e}=\frac{\pi }{2}\sqrt{\frac{E_{f}\cdot t_{f}\cdot s_{0}}{\tau_{b1}}}$$

Two proposals to calculate the model parameters of the bilinear local bond-slip law (Table 1) are indicated. Considering that debonding takes place in the concrete, the bond strength is determined by using the Mohr-Coulomb failure criterion, assuming that in a pure shear stress state, normal stresses are null. The differences between both proposals are:

- The dependence on the concrete strength in the formulation for the shear strength;
- The value of the maximum slip;
- The influence of the relationship between FRP and concrete surfaces, introduced by the shape factor *k_b_* (Equation (6)), where *b_f_* and *b* represent the widths of the FRP reinforcement and of the strengthened element, respectively.

(6) $$k_{b}=\sqrt{\frac{2-b_{f}/b}{1+b_{f}/b}}\geq 1$$

**Figure 2:**
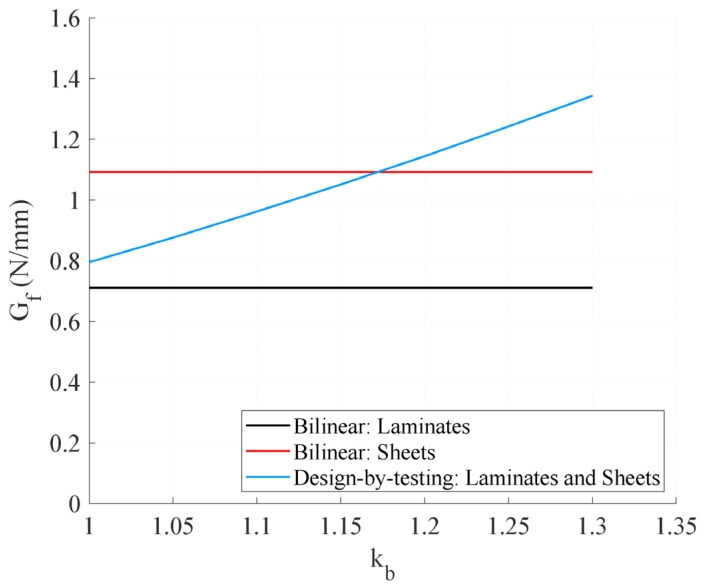
Fracture energy according to the suggested bond-slip approaches (values of *f_cm_* and *f_ctm_* equal to 44.76 MPa and 3.6 MPa, respectively).

The two approaches are compared in Figure 2 through the resulting fracture energy for a typical range of values of the factor *k_b_*. It can be observed that according to the approach from [33] *G_f_* is higher in specimens with sheet reinforcements than in those with strips. By using the approach from [34], *G_f_* increases proportionally to *k_b_*^2^, and, therefore, decreases proportionally to the ratio *b_f_*/*b*.

### 2.1. Simplified FRP Stress Limit Method (S1, S2)

This approach limits the ultimate FRP tensile stress at the most unfavourable section to a certain value *f_fb,IC,_* which depends on the end anchorage capacity of the FRP (Equation (3), considering *β_l_* equal to 1). In flexural applications, the transferred force in the FRP is higher than the end anchorage capacity due to the gradient of stresses in the FRP reinforcement. This phenomenon is considered through the calibration factor *k_cr_*, which has been defined with a value of 2.10 according to the assessment of the database described in [1].

(7) $$f_{fb,IC}=k_{cr}\cdot f_{fb}$$

Bilinear and design-by-testing approaches (Table 1) will be used for calculating *G_f_* and designated as S1 and S2, respectively.

### 2.2. More Accurate Method (MA1, MA2)

**Figure 3:**
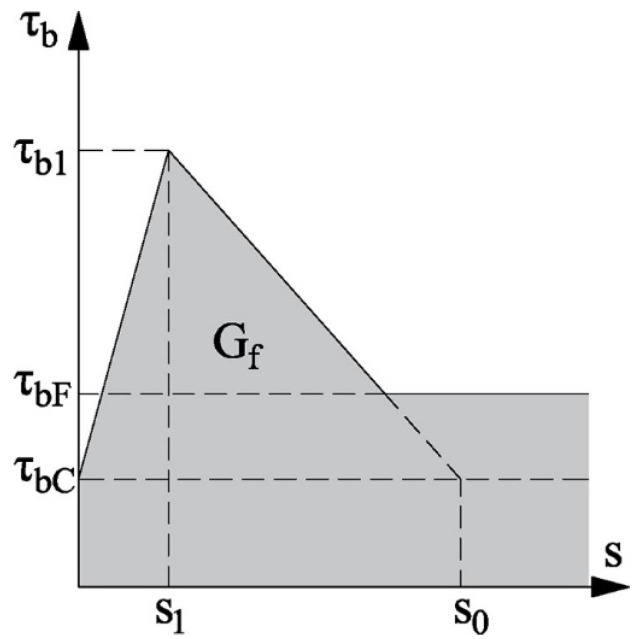
Modified bond law for the more accurate method.

The proposal of the German committee DAfStb was adopted, consisting of a more accurate method in which the maximum allowable increase in tensile force in the FRP reinforcement between two adjacent cracks is computed, considering a modified version of the bilinear bond law (Figure 3). According to the analysis of the experimental tests performed in [35,36], a significant increase in the bond force with respect to the typical bilinear bond law represented in Figure 1 was achieved, due to the contribution of friction (*τ_bF_*) and curvature components (*τ_bC_*).

The calculation of the maximum allowable increase in tensile force in the FRP (Δ*F_fR_*) can be performed in a detailed manner as in Equations (8)–(15) or in a simplified one as in Equation (22). Both formulations involve the calculation of FRP stresses at each crack, which implies the solution of equilibrium and compatibility equations at each iteration. Moreover, the resisting increment of force in the detailed analysis is dependent on the level of FRP force, changing at every crack and iteration, whereas in the simplified analysis, a unique value of Δ*F_fR_* needs to be computed, which was determined through a numerical approach to the more accurate method [22].

In the evaluation of the different methods, the detailed analysis will be addressed as MA1, whereas the simplified analysis will be MA2. Bilinear approach (Table 1) will be used for calculating the bond-slip parameters in both analyses.

#### 2.2.1. Detailed Analysis of FRP Force Difference (MA1)

As mentioned before, the bond resistance to change in the FRP force between cracks (Equation (8)) is the result of the addition of three components:

(8) $$\Delta F_{fR}=\Delta F_{f,B}+\Delta F_{f,F}+\Delta F_{f,C}$$

where Δ*F_f,B_* is the bond strength from the bilinear bond stress-slip curve, Δ*F_f,F_* is the term due to the frictional bond in the already debonded surface, and Δ*F_f,C_* is the contribution of the curvature of the member.

**Figure 4:**
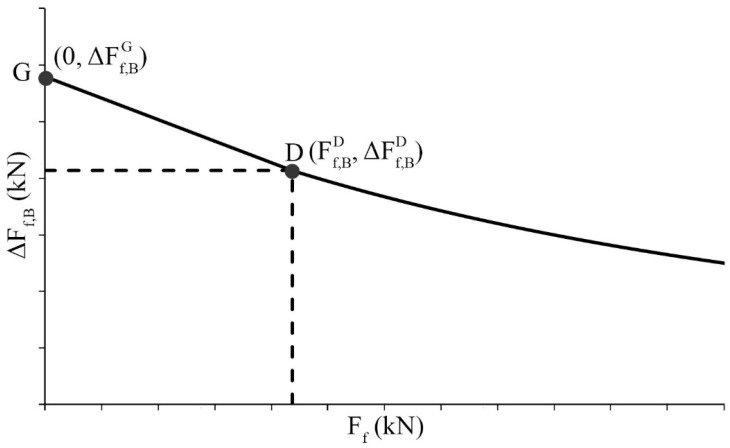
Maximum resisted increment of FRP force due to bond contribution.

The term related to the adhesive bond (Δ*F_f,B_*) is calculated using Equation (9). The first linear branch represents the cases where the stresses are not high enough to develop the whole bond stress-slip relationship in the crack spacing, as the value of *s*_0_ has not been attained (line G-D in Figure 4). Point G is obtained from Equation (10) assuming that the stress in the lower stressed crack is zero, and point D is calculated by Equations (11) and (12) when the required transfer length (*l_e_*) is equal to the crack spacing. The second branch of Equation (9) considers the cases in which *s*_0_ has already been attained (line beyond point D in Figure 4). In the latter, as a certain area of FRP reinforcement has detached, crack spacing becomes not relevant.

(9) $$\Delta F_{f,B}=\left\{\begin{array}{cc} \Delta F_{f,B}^{G}-\frac{\Delta F_{f,B}^{G}-\Delta F_{f,B}^{D}}{F_{f,B}^{D}}F_{f} & \mathrm{for}\;Ff\leq F_{f,B}^{D}\\ \sqrt{b_{f}^{2}\cdot \tau_{b1}\cdot s_{0}\cdot E_{f}\cdot t_{f}+F_{f}^{2}}-F_{f} & \mathrm{for}\;F_{f,B}^{D}<Ff<Ffu \end{array}\right.$$

where *F_fu_* is the ultimate force of the FRP reinforcement and:

(10) $$\Delta F_{f,B}^{G}=f_{fb}\left(s_{r}\right)\cdot b_{f}\cdot t_{f}$$

(11) $$\Delta F_{f,B}^{D}=\sqrt{b_{f}^{2}\cdot \tau_{b1}\cdot s_{0}\cdot E_{f}\cdot t_{f}+F_{f,B}^{D}{}^{2}}-F_{f,B}^{D}$$

(12) $$F_{f,B}^{D}=\frac{s_{0}\cdot E_{f}\cdot b_{f}\cdot t_{f}}{s_{r}}-\tau_{b1}\frac{s_{r}\cdot b_{f}}{4}$$

For this approach, according to [1], the term *f_fb_*(*s_r_*) is calculated through Equation (3), considering *l_b_* equal to *s_r_* and the bond parameters of approach [33] (Table 1).

The term related to the bond friction (Δ*F_f,F_*) is calculated using Equation (13). This term considers the friction that is produced in the surfaces where the debonding process starts, before the failure of the whole element between cracks.

When the tensile stress in the FRP is lower than $F_{f,B}^{D}$, the slip is lower than the maximum slip *s*_0_; therefore, debonding cannot happen, and friction is not produced (first branch of Equation (13)). After attaining the maximum slip, the friction stress developed in the debonded area will increase the bond strength. The debonded length is calculated by deducting from the crack spacing an effective length, derived from the bilinear bond law (second branch of Equation (13)).

(13) $$\Delta F_{f,F}=\left\{\begin{array}{cc} 0 & \mathrm{for}\;F_{f}\leq F_{f,B}^{D}\\ \tau_{bF}\cdot b_{f}\left[s_{r}-\frac{2t_{f}\cdot E_{f}}{\tau_{b1}}\cdot \left(\sqrt{\frac{\tau_{b1}\cdot s_{0}}{E_{f}\cdot t_{f}}+\frac{F_{f}^{2}}{b_{f}^{2}\cdot t_{f}^{2}\cdot E_{f}^{2}}}-\frac{F_{f}}{b_{f}\cdot t_{f}\cdot E_{f}}\right)\right] & \mathrm{for}\;F_{f,B}^{D}<F_{f}<F_{fu} \end{array}\right.$$

The bond friction strength is obtained by calibration from the experimental results [27]. In Equation (14), *α_cc_* considers long-term loading effects with a value between 0.8–1.0. In this study, *α_cc_* = 1.

(14) $$\tau_{bF}=17.5\alpha_{cc}\cdot f_{cm}^{0.89}$$

The term related to the contribution of the member curvature (Δ*F_f,C_*) is calculated using Equation (15). Due to the curvature of the beam caused by deflection, a pressure is applied onto the bottom of the concrete element so that the FRP can transfer higher stresses before debonding. Based on experimental tests [36], an empirical coefficient *κ_m_* = 33.3 × 10^3^ N/mm multiplies the curvature of the concrete element between cracks to compute the increment of transferred force due to the effect of curvature, where *ε_f_* and *ε_c_* are the strains in the FRP reinforcement and concrete at the lower stressed crack of the intermediate crack element, respectively.

(15) $$\Delta F_{f,C}=s_{r}\cdot \kappa_{m}\frac{\varepsilon_{f}-\varepsilon_{c}}{h}b_{f}$$

where tensile strains are considered positive and compressive strains, negative.

For determining the crack spacing at the ultimate limit state, the formulation in Equations (16)–(21) is proposed, where *l_e,_*_0_ is the transfer length of the reinforcing steel, *M_cr_* is the cracking moment, *z_s_* is the steel lever arm approximated to 0.85 *h*, *W_c,_*_0_ is the section modulus of the uncracked concrete gross section, *F_bsm_* is the bond force per unit length, *n_s,i_* is the number of steel rebars with diameter *ø_s,i_*, *f_bsm_* is the mean bond stress, and the parameters *κ_νb_*_1_ and *κ_νb_*_2_ can be assumed equal to 1.0 for good bond conditions and *κ_νb_*_1_ = 0.7 and *κ_νb_*_2_ = 0.5 for medium bond conditions.

(16) $$s_{r}=1.5l_{e,0}$$

(17) $$l_{e,0}=\frac{M_{cr}}{z_{s}\cdot F_{bsm}}$$

(18) $$M_{cr}=\kappa_{fl}\cdot f_{ctm}\cdot W_{c,0}$$

(19) $$\kappa_{fl}=\left(1.6-\frac{h}{1000}\right)\geq 1$$

(20) $$F_{bsm}=\sum_{i=1}^{n}n_{s,i}\pi \cdot \emptyset_{s,i}\cdot f_{bsm}$$

(21) $$f_{bsm}=\left\{\begin{array}{cc} 0.43\kappa_{\nu b1}\cdot f_{cm}^{2/3} & for\;ribbed\;bars\\ 0.28\kappa_{\nu b2}\cdot \sqrt{f_{cm}} & for\;ribbed\;bars \end{array}\right.$$

#### 2.2.2. Simplified Analysis of FRP Force Difference (MA2)

Based on a numerical analysis from the previous detailed procedure [22], a constant value for the increment of resisting the tensile force of each element between cracks is proposed. This formulation, also accounting for the three terms related to bond resistance, friction, and curvature is deemed to be more conservative than the detailed approach, providing a simpler methodology and reducing the computational effort.

(22) $$\Delta F_{fRm}=\left(1.84\tau_{b1m}\sqrt{s_{r}}+0.095\tau_{bFm}\cdot s_{r}^{4/3}+\frac{\kappa_{h}}{h}\cdot s_{r}^{1/3}\right)b_{f}$$

where *κ_h_* = 2739, *s_r_* is the crack spacing, limited to 400 mm, and *h*, the member height, is greater than 100 mm. The maximum strain in the FRP reinforcement should not exceed the value of 0.01.

## 3. Experimental Campaign

### 3.1. Layout and Instrumentation

**Figure 5:**
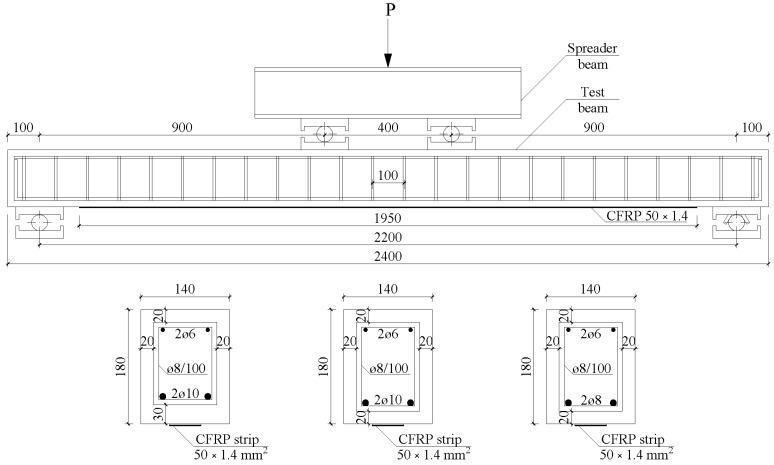
Specimen details (dimensions in mm).

An experimental campaign was performed to assess the different approaches presented above. Three CFRP-strengthened RC beams from two different series were tested under a four-point bending configuration (Figure 5). The differences between series I and II were the concrete strength (properties are reported in Table 2) and the concrete cover.

The dimensions of the specimens are shown in Figure 5. The total length of the beams was 2400 mm, with a distance between supports of 2200 mm and a shear span of 900 mm. The beams were designed with a relatively high ratio between the shear span and the effective depth to induce failure by ICD [41]. All beams had a cross-section of 140 × 180 mm. The tensile steel reinforcement consisted of two bars of diameter 8 mm or 10 mm (depending on the beam, see Figure 5) and two bars of diameter 6 mm in the compression zone to hold the internal shear reinforcement. The latter consisted of steel stirrups of diameter 8 mm with a spacing of 100 mm placed along the beam length. All beams were externally strengthened with a CFRP laminate of 50 × 1.4 mm. The properties of the steel and CFRP are reported in Table 2.

The specimens were designated as EBR-X-dY, where X is the series (I or II) and Y is the diameter of steel tensile reinforcement (8 mm or 10 mm).

To ensure a sufficient bonding between the concrete and the CFRP laminate, the outer layer of concrete in all beams was removed by bush-hammering the surface and then cleaned with compressed air. After this procedure, a thin layer of a two-component epoxy resin was applied onto the CFRP laminate, which was immediately placed on the concrete surface. The adhesive used in this study was S&P Resin 220 HP, a thixotropic and solvent-free adhesive. Its properties after a curing time of 7 days, according to the manufacturer data sheet, are reported in Table 2. The specimens were cured for 135 days, in the case of series I, and 37 days in the case of series II, in laboratory conditions.

The test was performed under a displacement-controlled mode with a rate of 0.60 mm/min. The load was applied by a hydraulic jack to a spreader beam, which transmitted the load to the beam specimens within the span of 400 mm.

A 200-kN load cell was placed under the actuator to measure the applied force. Three linear variable displacement transducers (LVDT) were placed to measure the mid-span deflection: one LVDT was located at the central section of the beam, and the other two were located at each one of the two supports. The concrete strain at the mid-span was measured in beams of series II using two strain gauges (SG): one at the concrete top section and another at 2 cm from the top, attached to the beam face. The CFRP strain at the mid-span was measured using one SG attached to the CFRP surface.

### 3.2. Experimental Results

**Figure 6:**
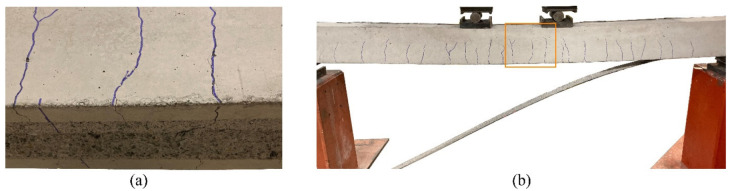
Failure of beam EBR-I-d10: (**a**) Failure surface and (**b**) ICD failure and crack pattern.

The values of the load, mid-span deflection, and CFRP strain and concrete strain at failure are reported in Table 3. All beams failed by the intermediate crack debonding of the laminates within a thin layer of the concrete surface (Figure 6a) initiated in the central zone, where the widest cracks were identified (Figure 6b).

Analysing the experimental values of maximum CFRP and concrete strains, the premature nature of the debonding failure mode can be observed. Considering the ultimate CFRP strain of 16 × 10^–3^ according to the manufacturer, the tested laminates were working at between 30% and 40% of their tensile capacity. As for the concrete, the maximum compressive strain in series II was approximately 50% of the design compressive strain (3.5 × 10^–3^) [42]. The maximum concrete strain in specimen EBR-I-d10 could not be recorded due to damage in the corresponding SG during the test.

**Figure 7:**
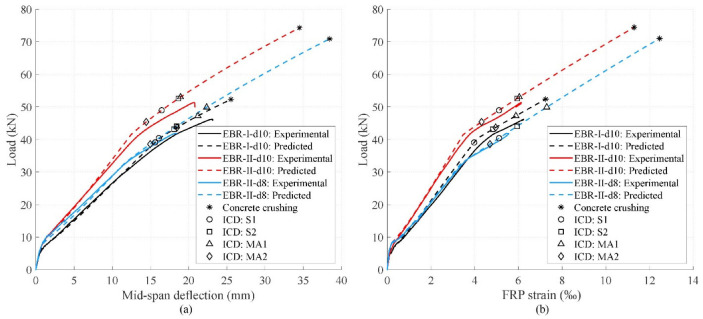
Behaviour of tested specimens: (**a**) load-deflection curves and (**b**) load-strain curves.

Experimental load versus mid-span deflection and maximum CFRP strain are represented in Figure 7a,b, respectively, along with the theoretical predictions. The theoretical behaviour was calculated considering force equilibrium and strain compatibility in the cross-section. The EC-2 [42] parabola-rectangle diagram for concrete (*ε_c_*_0_ = 2‰ and *ε_cu_* = 3.5‰) and a bilinear diagram for steel were considered. The shrinkage effect was taken into account in the calculations through a value of the shrinkage strain of 2.30 × 10^–4^, gathered from the characterisation of the concrete, following the methodology of [42]. The values of the ICD failure load predictions were calculated according to the approaches presented above. As the interest of this study was to compare the theoretical with the experimental results, mean values were considered; therefore, strength reduction factors were not taken into account (*γ_c_* = *γ_s_* = *γ_f_* = *γ_fb_* = 1).

As was expected, all beams followed a trend with three branches defined by the cracking, yielding, and failure loads. The predicted behaviour agreed well with the experimental one. In all specimens, ICD took place after the yielding of the internal tensile steel reinforcement, which is one of the desirable flexural failure modes [1]. Debonding in series I occurred near the theoretical concrete crushing failure load, whereas in series II, the difference between the debonding and theoretical concrete crushing was much higher. This shows that a higher concrete strength, which is the case of the beams in series II, improved the bending capacity of the beam, yet concluded in a higher underutilisation of the CFRP reinforcement.

The values of the CFRP strain at failure were similar among specimens EBR-I-d10 and EBR-II-d10, despite the differences in concrete strength. However, specimen EBR-II-d8, with a lower steel reinforcement ratio but higher concrete strength than EBR-I-d10, achieved a lower maximum value of CFRP strain. This implies that a higher amount of internal tensile steel reinforcement was more effective in terms of the use of FRP reinforcement than a high concrete compressive strength. It should be noted that the experimental CFRP strain in Figure 7b corresponds to the SG that gave the maximum value in each beam, which, due to the nature of crack patterns, was in a different location depending on the specimen. The appearance of cracks in the tensile face of the beam may change the CFRP strain distribution, which inherently implies a certain lack of precision in capturing the maximum value along the laminate.

Experimental failure load and CFRP strain have been compared with the predicted values in Table 4. For this experimental campaign, values of ICD load are well predicted by all approaches, with a mean experimental-to-theoretical ratio between 0.93 and 1.09 and a coefficient of variation (CoV) between 0.03 and 0.08. The variation in the CFRP strain values is higher, probably due to the difficulty of capturing the maximum value as mentioned above.

Approaches S1 and MA2 are shown to be the most conservative, with a mean experimental-to-predicted failure load ratio of 1.09. On the other hand, MA1 has been the least conservative approach (0.93), over-predicting the ICD load in terms of average values.

The parameters of the bond law used for the predictions in the different approaches are reported in Table 5. When using [34], higher values of the maximum shear stress and ultimate slip are attained, resulting in a higher fracture energy than in the former. This is the reason why, comparing both simplified approaches, S1 is more conservative than S2.

The bonded length is not taken into account in the simplified methods as it is considered that the effective length, and, therefore, the full anchorage capacity, are attained between the point of maximum strain in the CFRP laminate and the support. On the contrary, in MA1, the effective length is compared with the crack spacing, using the most restricting value of both for the calculations. In MA2, the value of the crack spacing is used without considering the effective length. This means that in cases where the crack spacing is lower than the effective length (reducing the anchorage capacity of the CFRP reinforcement according to Equations (3) and (4)), as in beams EBR-I-d10 and EBR-II-d10, the bond strength considered in the accurate approaches is lower than in the simplified ones. However, due to the contribution of the bond friction and beam curvature, MA1 is still less conservative than the simplified approaches. Regarding MA2, as it is developed from a simplification of the detailed model MA1 on the safe side, it gives similar experimental-to-predicted failure load ratios as S1 and S2.

## 4. Models Assessment

### 4.1. Database

A database of 65 FRP-strengthened RC beams that failed by ICD [43,44,45,46,47,48,49,50,51,52,53,54,55,56,57,58,59] has been analysed, in addition to the 3 specimens described in the previous section, thus giving a total of 68 specimens. The geometrical and material properties of the beams are listed in Table 6, as well as the debonding failure load (*P_u,exp_*) and bending moment (*M_u,exp_*). The specimens were externally strengthened with bonded CFRP reinforcement; 36 (55%) of them were pre-cured, and 30 (45%) of them were wet laid-up. Most of the beams were tested under three- or four-point bending tests, the most common configuration found in the literature due to the simplicity of the test and the advantage of being able to separate moment and shear failure modes, with the exception of specimens LP4SP1000 and LP8SP1000 [55], which were tested under six- and ten-point bending configurations, respectively. With the purpose of having the same conditions in all specimens in the database, none of them had anchorages.

All beams have rectangular sections with a geometry defined by the distance between supports (*L_beam_*), the shear span (*L_shear_*), the sectional width (*b*) and total depth (*h*), the effective depth (*d*), the tensile and compression steel reinforcement (*A_s1,_ A_s2_*), and the CFRP width (*b_f_*), thickness (*t_f_*) and type (*P* for pre-cured and *W* for wet lay-up).

Concrete properties are given by the mean concrete compressive strength (*f_cm_*) and the mean tensile strength (*f_ctm_*). The values of *f_cm_* range between 16.8 MPa and 55.8 MPa. Where *f_ctm_* is not defined, it has been computed as specified in the *fib* Model Code [60] (Equation (23)). Steel properties are given by the yielding strength (*f_y_*) and the elastic modulus (*E_s_*). Where *f_y_* and *E_s_* were not defined, 500 MPa and 200 MPa have been considered, respectively. CFRP properties are given by the tensile strength (*f_fu_*), the ultimate strain (*ε_fu_*), and the elastic modulus (*E_s_*). According to [1], in cases of wet lay-up systems where the geometrical and material properties of the CFRP sheet were not specifically detailed, only the properties of the fibres in its cross-section have been considered. The properties of the adhesive are not listed as they are not used in the calculations.

(23) $$f_{ctm}=0.3{\left(f_{cm}-8\right)}^{2/3}$$

### 4.2. Discussion

**Figure 8:**
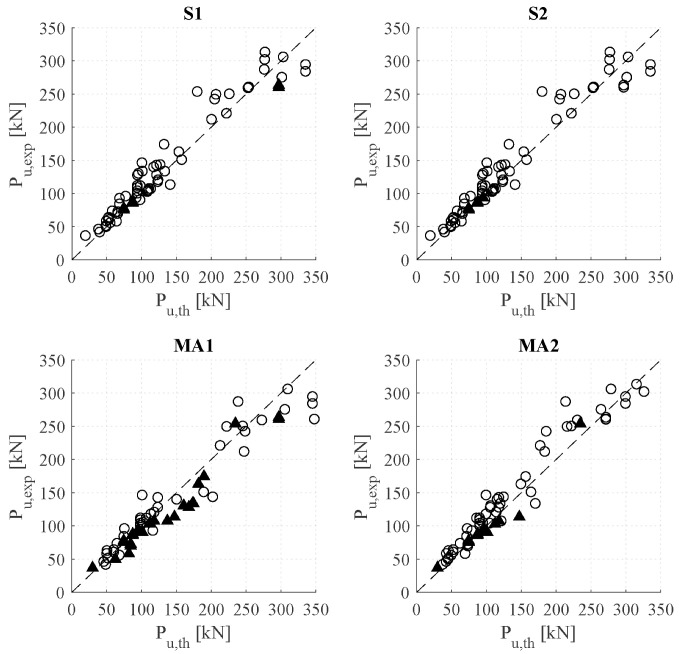
Predicted versus experimental failure load for the analysed beams. Legend: ○ Theoretical failure mode is ICD. ▲Theoretical failure mode is concrete crushing (CC)/fibre rupture (FR).

Predictions based on the models described in the previous section are reported in Table 7 for all beams in the database. The results are presented in terms of failure load (*P_u,th_*) and experimental-to-predicted failure load ratio (*P_u,exp_*/*P_u,th_*). Furthermore, the theoretical location of the initiation of debonding (*x_failure_*), as well as the tensile force in the FRP at failure (*F_fR_*) for the simplified methods and the increment of force for the accurate ones (∆*F_fR_*) are also reported in Table 7 as further elements of comparison. The mean values of the ratio *P_u,exp_*/*P_u,th_*, standard deviation (StDev), and CoV are shown in Table 8. Moreover, theoretical predictions and experimental results are presented in Figure 8. White circles represent theoretical ICD failure, while black triangles represent beams that, theoretically, should have arrived at their rupture load (either concrete or FRP) before ICD.

For the beams in the present database, the values of the ICD load are well predicted by all approaches, with a mean ratio of *P_u,exp_*/*P_u,th_* between 0.96 and 1.10 and a CoV between 0.12 and 0.17. As was also observed in the experimental campaign, MA1 is the least conservative approach in terms of average values (0.96), whereas S2 is the second least conservative one (1.04). The latter, although being a very simple method, presents highly accurate results. The most conservative methods in this study were S1 and MA2, with an experimental-to-predicted failure load ratio of 1.09.

#### 4.2.1. Analysis of the Location of Initiation of Debonding

Regarding the location of the theoretical initiation of debonding, for the beams analysed in this study under three- or four-point bending configurations, the maximum values of bending moments and shear forces are located under the load application point. Considering this, jointly with a constant increment of the FRP force along the FRP in the shear span due to a constant shear force, the load application point becomes the critical point in the beam, as it can be observed from the *x_failure_* values. For this reason, the ratios between the experimental and predicted failure loads are similar within the different approaches. However, the critical point might be shifted from the central zone to the shear span due to a higher increment of force in the FRP caused by the yielding of the steel. This effect will only be appreciated by the more accurate (or stress increment-based) approaches, which is the reason for the difference in *x_failure_* values between those and the simplified approaches.

If a more distributed load is applied (common case in practice), the relationship between the bending moment and the shear force would change. The maximum moment region would be located at the same point, leading to the same predictions when considering the simplified (or strain-based) approaches. However, the shear and increment of the FRP force would not be constant, being lowest at the maximum moment region and highest at the support, leading to higher ICD load predictions according to the more accurate methods. In that scenario, the differences between simplified and more accurate methods would be greater, the simplified approaches being more conservative than the accurate ones, as was also observed in [29].

#### 4.2.2. Analysis of Simplified Methods

Similar to the beams in the experimental campaign, S1 predictions were more conservative than in S2 for the specimens in the database strengthened with pre-cured laminates. The reason for this is that, theoretically, a higher bond fracture energy (*G_f_*) was attained considering the formulation in [34] (used in S2) instead of [33] used in S1. On the contrary, in specimens reinforced with wet lay-up sheets, the fracture energy and ICD predictions in S1 were higher than in S2. By analysing the results, the reason of this fact was found to be the shape factor *k_b_*, affecting the bond parameters in the formulation in [34].

#### 4.2.3. Analysis of More Accurate Methods

Analysing the contribution of bond, friction, and curvature in the more accurate methods, it can be observed that in MA1, friction was the least contributing factor in all specimens. Regarding the other components, specimens with high CFRP reinforcement ratios and/or high concrete strength had a high contribution of the bond component but lower curvature contribution, whereas specimens with low values of those properties had the opposite effect. As MA2 is developed from a simplification on the safe side of the MA1 model with calibration factors, this tendency was not that clear.

When one or more of the conditions listed below are met, the predicted failure mode might be concrete crushing (CC) or FRP rupture (FR) instead of ICD, although beams experimentally failed by ICD. As reported in Table 8, this mostly happened when using the MA1 approach, in which only 63% of the specimens theoretically failed by ICD. However, it was observed that the experimental-to-predicted failure load ratios considering the specimens that theoretically failed by CC or FR (case 1 in Table 8) were similar to those not considering them (case 2 in Table 8). Some possible reasons for this difference in the failure mode prediction could be the following:

- The similarity of the experimental failure load due to ICD with the predicted ultimate capacity of the beam without considering debonding;
- The value of the concrete strain, which may have exceeded the ultimate value of 3.5 ‰ considered in this study;
- The difficulty of finding the exact position of the cracks and the point where failure initiates;
- The differences in the experimental and predicted parameters of the bond law;
- A combination of these with other factors such as concrete strength, steel, and CFRP reinforcement ratio and the geometry of the beam.

## 5. Conclusions

In the present study, the formulation published in the *fib* Bulletin 90 [1] regarding the intermediate crack debonding (ICD) has been assessed through comparison with a database of 68 RC beams strengthened with EB CFRP. Moreover, an experimental campaign of three beams tested under a four-point bending configuration was carried out to have a better understanding of the ICD failure mode, as well as to be able to compare the results with the theoretical predictions in more detail. Based on these results, the following conclusions can be drawn:

- In all specimens, ICD took place after the yielding of the internal tensile steel reinforcement, corresponding to the desirable flexural failure modes. The results showed that a higher concrete strength improved the bending capacity of the beam (series II-44.8 MPa vs. series I-23.9 MPa), yet it was much lower than the theoretical prediction in the absence of debonding. Likewise, a higher amount of steel reinforcement led to a higher bending capacity with similar CFRP strains at failure;
- The values of the ICD load were well predicted by all approaches, with a mean experimental-to-predicted failure load ratio between 0.96 and 1.09 and a CoV between 0.12 and 0.17;
- Predictions more similar to the experimental results were obtained with the more accurate method MA1 (0.96) approach in comparison with the MA2 (1.09) and simplified methods S1 (1.09) and S2 (1.04), although with more computational effort. However, for the beams in the database following the usual test configurations with a three- or four-point bending configuration, the differences between the methods were low;
- The value of the bond fracture energy affects the ICD load. For this database, the formulations used in S2 considering the shape factor kb were slightly more accurate predictions than S1.

## Figures and Tables

**Table 1:** Suggested mean values for the parameters in the bilinear bond law [33,34].

Bond-Slip Approach	Reinforcement Type	*G_f_* (MPa·mm)	*τ_b_*_1_ (MPa)	*s*_0_ (mm)
Bilinear [33]	Laminates	0.056$\sqrt{f_{cm}\cdot f_{ctm}}$	0.530$\sqrt{f_{cm}\cdot f_{ctm}}$	0.210
	Sheets	0.086$\sqrt{f_{cm}\cdot f_{ctm}}$	0.720$\sqrt{f_{cm}\cdot f_{ctm}}$	0.240
Design-by-testing [34]	Laminates and sheets	0.063$k_{b}^{2}{\cdot f}_{cm}^{2/3}$	0.500$k_{b}^{2}{\cdot f}_{cm}^{2/3}$	0.250

Note: *f_cm_* and *f_ctm_* are the mean compressive and tensile strength of the concrete, respectively.

**Table 2:** Material properties.

Material	Concrete Age at Test Day (Days)	Compressive Strength (MPa)	Yielding Strength (MPa)	Ultimate Tensile Strength (MPa)	Yielding Strain (‰)	Ultimate Tensile Strain (‰)	Elastic Modulus (GPa)
Concrete series I	28	23.88 ^1^	–	2.46 ^2^	–	–	29.31 ^3^
Concrete series II	46	44.76 ^1^	–	3.60 ^2^	–	–	36.21 ^3^
Steel			569.96 ^4^		2.73 ^4^		209.76 ^4^
CFRP laminate		–	–	2800 ^5^	–	16 ^5^	170 ^5^
Resin 220 HP		–	–	15 ^5^	–	–	7.1 ^5^

^1^ Determined according to [37]. ^2^ Determined according to [38]. ^3^ Determined according to [39]. ^4^ Determined according to [40] ^5^ Provided by the manufacturer (S&P).

**Table 3:** Experimental results.

Series	Specimen Label	*P_u_* (kN)	*δ_u_* (mm)	*ε_fu_* (‰)	*ε_cu_* (‰)	Failure Mode
I	EBR-I-d10	46.19	23.11	6.25	– ^1^	IC
II	EBR-II-d10	51.35	20.76	6.14	–1.70	IC
	EBR-II-d8	41.89	18.40	5.04	–1.77	IC

^1^ SG damaged during the test.

**Table 4:** Experimental results versus theoretical predictions for tested beams.

Specimen Label	S1		S2		MA1		MA2	
	*ε_fu,exp_* */ε_fu,th_*	*P_u,exp_* */P_u,th_*	*ε_fu,exp_* */ε_fu,th_*	*P_u,exp_* */P_u,th_*	*ε_fu,exp_* */ε_fu,th_*	*P_u,exp_* */P_u,th_*	*ε_fu,exp_* */ε_fu,th_*	*P_u,exp_* */P_u,th_*
EBR-I-d10	1.57	1.18	1.30	1.07	1.06	0.98	1.26	1.06
EBR-II-d10	1.20	1.05	1.03	0.98	1.02	0.97	1.42	1.13
EBR-II-d8	0.99	1.04	0.85	0.95	0.69	0.84	1.07	1.09
Mean (-)	1.25	1.09	1.06	1.00	0.92	0.93	1.25	1.09
StDev (-)	0.30	0.08	0.23	0.06	0.20	0.08	0.18	0.04
CoV (-)	0.24	0.07	0.21	0.06	0.22	0.08	0.14	0.03

**Table 5:** Theoretical parameters of the bond law used in the different approaches.

Specimen Label	*s_r_* (mm)	S1, MA1, MA2 [33]				S2 [34]			
		*l_e_* (mm)	*G_f_* (MPa·mm)	*τ_b_*_1_ (MPa)	*s*_0_ (mm)	*l_e_* (mm)	*G_f_* (MPa·mm)	*τ_b_*_1_ (MPa)	*s*_0_ (mm)
EBR-I-d10	115.56	173.75	0.43	4.08	0.21	171.03	0.63	5.02	0.25
EBR-II-d10	111.37	133.73	0.72	6.90	0.21	138.71	0.95	7.63	0.25
EBR-II-d8	139.22	133.73	0.72	6.90	0.21	138.71	0.95	7.63	0.25

**Table 6:** Geometrical and material properties for the specimens in the database.

Specimens	*L_beam_*	*L_shear_*	*b*	*h*	*d*	*A_s1_*	*A_s2_*	*b_f_* × *t_f_*	CFRP Type	*f_cm_*	*f_ctm_*	*f_y_*_1_	*f_y_*_2_	*E_s_*_1_	*E_s_*_2_	*f_fu_*	*ε_fu_*	*E_f_*	*P_u,exp_*	*M_u,exp_*
	mm	mm	mm	mm	mm	mm	mm	mm × mm	^1^	MPa	MPa	MPa	MPa	GPa	GPa	MPa	‰	GPa	kN	kNm
Al-Saawani et al. (2015) [58]																				
S-0.5-35-240	3000	1000	400	250	215	3ø14	3ø10	240 × 1.4	P	35.3	2.7	475	533	200	207	2800	17	165	211.9	106.0
S-0.9-35-240	3000	1000	400	250	215	5ø14	4ø10	240 × 1.4	P	35.3	2.7	475	533	200	207	2800	17	165	259.3	129.7
S-0.9-24-240	3000	1000	400	250	215	5ø14	4ø10	240 × 1.4	P	23.6	1.9	475	533	200	207	2800	17	165	250.3	125.2
S-0.9-17-240	3000	1000	400	250	215	5ø14	4ø10	240 × 1.4	P	17.4	1.3	475	533	200	207	2800	17	165	249.6	124.8
S-1.3-35-240	3000	1000	400	250	215	7ø14	4ø10	240 × 1.4	P	35.3	2.7	475	533	200	207	2800	17	165	306.1	153.1
SN-0.9-35-240	5000	1640	250	400	362	4ø16	2ø10	240 × 1.4	P	35.3	2.7	450	533	190	207	2800	17	165	260.8	213.9
Al-Zaid et al. (2014) [59]																				
B-0.6-0	5000	2000	500	250	204	4ø16	4ø12	480 × 1.4	P	30.0	2.4	562	533	205	207	2800	17	165	253.8	253.8
B-0.3-0	5000	2000	500	250	204	4ø16	4ø12	240 × 1.4	P	30.0	2.4	562	533	205	207	2800	17	165	174.2	174.2
Aram et al. (2008) [50]																				
B3	2000	667	250	150	120	3ø8	3ø8	50 × 1.2	P	49.0	3.6	485	485	200 ^2^	200 ^2^	2000	9	214	62.8	20.9
B4	2000	667	250	150	120	3ø8	3ø8	50 × 1.2	P	52.0	3.7	485	485	200 ^2^	200 ^2^	2700	17	155	58.4	19.5
Bilotta et al. (2015) [53]																				
EBR_c_1.4×40_1	2100	925	120	160	115	2ø10	2ø10	40 × 1.4	P	16.8	1.3	540	540	200 ^2^	200 ^2^	2052	12	171	36.5	16.9
El-Zeadani (2019) [54]																				
CFRP-B1	3500	1750	200	300	254	2ø16	2ø12	50 × 1.2	P	22.7	2.8	535	535	154	154	2800	17	165	84.3	73.8
Fu et al. (2017) [56]																				
B1S1	3600	1300	200	450	395	3ø16	2ø16	145 × 0.67	W	49.0	3.6	531	531	214	214	3263	13	251	275.4	179.0
B1S2	3600	1300	200	450	395	2ø16	2ø16	145 × 0.67	W	25.9	2.1	525	525	206	206	3263	13	251	242.4	157.6
Fu et al. (2018) [55]																				
LP2SP1750	4000	1750	200	450	395	2ø16	2ø16	111 × 1.0	W	47.0	3.5	431	431	200 ^2^	200 ^2^	4654	18	258	151.1	132.2
LP2SP1250	4000	1250	200	450	395	2ø16	2ø16	112 × 1.0	W	47.1	3.5	431	431	200 ^2^	200 ^2^	4654	18	258	221.0	138.1
LP2SP1000	4000	1000	200	450	395	2ø16	2ø16	110 × 1.0	W	48.2	3.5	431	431	200 ^2^	200 ^2^	4654	18	258	287.2	143.6
LP4SP1000	4000	1000	200	450	395	2ø16	2ø16	110 × 1.0	W	48.5	3.5	431	431	200 ^2^	200 ^2^	4654	18	258	302.2	151.1
LP8SP1000	4000	1000	200	450	395	2ø16	2ø16	110 × 1.0	W	48.5	3.5	431	431	200 ^2^	200 ^2^	4654	18	258	313.4	151.6
Hong (2012) [45]																				
BPS60	3000	1500	200	300	270	3ø10	3ø13	50 × 1.3	P	20.7	2.2	475	466	201	211	2412	13	180	64.2	48.2
BPS90	3000	1500	200	300	270	3ø10	3ø13	50 × 1.3	P	20.7	2.2	475	466	201	211	2412	13	180	60.5	45.4
BPD90	3000	1500	200	300	270	3ø10	3ø13	50 × 2.6	P	20.7	2.2	475	466	201	211	2412	13	180	73.7	55.3
BPDW90	3000	1500	200	300	270	3ø10	3ø13	100 × 1.3	P	20.7	2.2	475	466	201	211	2412	13	180	92.9	69.7
Kotynia et al. (2009) [52]																				
B-08S	4200	1400	150	300	269	3ø12	2ø10	50 × 1.2	P	32.3	2.8	490	524	195	290	2915	17	172	96.0	67.2
B-08M	4200	1400	150	300	269	3ø12	2ø10	120 × 1.4	P	37.3	3.5	490	524	195	290	2743	12	220	140.0	98.0
Maalej et al. (2005) [44]																				
A3	1500	500	115	146	120	3ø10	2ø10	108 × 0.17	W	39.8	3.41	547	247	180	180	3550	15	235	77.5	19.4
A4	1500	500	115	146	120	3ø10	2ø10	108 × 0.17	W	39.8	3.41	547	247	180	180	3550	15	235	75.5	18.9
A5	1500	500	115	146	120	3ø10	2ø10	108 × 0.33	W	39.8	3.41	547	247	180	180	3550	15	235	87.4	21.9
A6	1500	500	115	146	120	3ø10	2ø10	108 × 0.33	W	39.8	3.41	547	247	180	180	3550	15	235	77.5	19.4
B3	3000	1000	230	292	240	3ø20	2ø20	216 × 0.33	W	39.8	3.41	544	544	183	183	3550	15	235	75.5	18.9
B4	3000	1000	230	292	240	3ø20	2ø20	216 × 0.33	W	39.8	3.41	544	544	183	183	3550	15	235	87.4	21.9
B5	3000	1000	230	292	240	3ø20	2ø20	216 × 0.66	W	39.8	3.41	544	544	183	183	3550	15	235	85.8	21.5
B6	3000	1000	230	292	240	3ø20	2ø20	216 × 0.66	W	39.8	3.41	544	544	183	183	3550	15	235	263.5	131.8
C3	4800	1600	368	467	384	3ø32	2ø32	368 × 0.50	W	41	3.24	552	552	181	181	3550	15	235	260.3	130.2
C4	4800	1600	368	467	384	3ø32	2ø32	368 × 0.50	W	41	3.24	552	552	181	181	3550	15	235	294.7	147.4
C5	4800	1600	368	467	384	3ø32	2ø32	368 × 0.99	W	41	3.24	552	552	181	181	3550	15	235	284.3	142.2
Niu et al. (2006) [51]																				
A1	4200	2100	960	203	173	3ø19	–	200 × 1.28	P	31.6	2.5	452	–	192	–	2446	13	184	127.8	134.2
A2	4200	2100	960	203	173	3ø19	–	200 × 1.21	P	33.4	2.6	452	–	192	–	2384	12	195	130.4	136.9
A3	4200	2100	960	203	173	3ø19	–	300 × 1.35	W	35.2	2.7	452	–	192	–	724	9	80	102.7	107.8
A4	4200	2100	960	203	173	3ø19	–	300 × 2.55	W	34.4	2.7	452	–	192	–	859	8	109	133.7	140.4
A5	4200	2100	960	203	173	3ø19	–	200 × 2.55	W	35.9	2.8	452	–	192	–	859	8	109	107.4	112.8
A6	4200	2100	960	203	173	3ø19	–	200 × 1.35	W	35.1	2.7	452	–	192	–	724	9	80	93.7	98.4
B1	4200	1600	960	203	173	3ø19	–	200 × 1.28	P	35.2	2.7	452	–	192	–	2446	13	184	143.7	115.0
B2	4200	1600	960	203	173	3ø19	–	300 × 1.35	W	34.5	2.7	452	–	192	–	724	9	80	113.4	90.7
C2	4200	2100	960	203	177	7ø13	–	200 × 1.28	P	33.4	2.6	446	–	196	–	2446	13	184	133.8	140.5
C3	4200	2100	960	203	177	7ø13	–	300 × 1.35	W	34.1	2.6	446	–	196	–	724	9	80	107.2	112.6
C4	4200	2100	960	203	177	7ø13	–	200 × 1.35	W	34.5	2.7	446	–	196	–	724	9	80	90.5	95.0
Oller (2005) [43]																				
1D2	2000	1000	300	200	160	2ø16	2ø8	100 × 1.4	P	35.2	2.8	500	500	200 ^2^	200 ^2^	2500	17	150	112.0	56.0
1C1	2000	1000	300	200	160	2ø16	2ø8	100 × 1.4	P	35.2	2.8	500	500	200 ^2^	200 ^2^	2500	17	150	104.0	52.0
1B1	2000	1000	300	200	160	2ø16	2ø8	100 × 1.4	P	35.2	2.8	500	500	200 ^2^	200 ^2^	2500	17	150	100.4	50.2
1A	2000	1000	300	200	160	2ø16	2ø8	100 × 1.4	P	35.2	2.8	500	500	200 ^2^	200 ^2^	2500	17	150	109.0	54.5
2D1	2000	1000	300	200	158	2ø20	2ø8	100 × 1.4	P	35.2	2.8	500	500	200 ^2^	200 ^2^	2500	17	150	128.0	64.0
2D2	2000	1000	300	200	158	2ø20	2ø8	200 × 1.4	P	35.2	2.8	500	500	200 ^2^	200 ^2^	2500	17	150	163.0	81.5
2C1	2000	1000	300	200	158	2ø20	2ø8	100 × 1.4	P	35.2	2.8	500	500	200 ^2^	200 ^2^	2500	17	150	142.8	71.4
Peng et al. (2014) [47]																				
PRS-EB	3300	1300	150	350	304	2ø16	2ø22	50 × 1.2	P	21.3	1.7	500 ^2^	500 ^2^	200 ^2^	200 ^2^	3100	19	165	146.4	95.2
Rusinowski and Täljsten (2009) [49]																				
Beam 2	1900	950	120	170	135	2ø10	2ø10	100 × 1.4	P	55.8	3.8	678	678	200 ^2^	200 ^2^	2000	13	155	72.6	34.5
Beam 6	1900	950	120	170	135	2ø10	2ø10	100 × 1.4	P	55.8	3.8	678	678	200 ^2^	200 ^2^	2000	13	155	69.7	33.1
Slaitas and Valivonis (2020) [57]																				
BS-0	2810	955	180	300	263	2ø14	2ø10	50 × 1.2	P	50.0	3.6	569	538	200	200	2628	15	170	120.7	57.6
Turco et al. (2017) [48]																				
BL_A-w	1524	762	152	305	262	3ø10	2ø10	64 × 0.89	W	27.3	2.2	490	490	200	200	1394	16	87	111.8	42.6
BL_2A-w	1524	762	152	305	262	3ø10	2ø10	64 × 1.78	W	27.3	2.2	490	490	200	200	1394	16	87	105.2	40.1
BH_2A-w	1524	762	152	305	262	3ø10	2ø10	64 × 1.78	W	42.6	3.2	490	490	200	200	1394	16	87	117.9	44.9
SL_A-w	1524	762	356	152	129	3ø10	–	64 × 0.89	W	27.3	2.2	490	–	200	–	1394	16	87	49.9	19.0
SL_2A-2w	1524	762	356	152	129	3ø10	–	127 × 0.89	W	27.3	2.2	490	–	200	–	1394	16	87	58.1	22.1
SL_2A-w	1524	762	356	152	129	3ø10	–	64 × 1.78	W	27.3	2.2	490	–	200	–	1394	16	87	55.9	21.3
Zhou et al. (2020) [46]																				
B2	3000	1200	250	300	267	2ø16	2ø10	100 × 0.17	W	28.5	2.2	538	401	200	210	3319	14	230	104.0	62.4
Present study																				
EBR-I-d10	2200	900	140	180	137	2ø10	2ø6	50 × 1.4	P	23.9	2.5	570	570	209	209	2800	16	170	46.2	20.8
EBR-II-d10	2200	900	140	180	147	2ø10	2ø6	50 × 1.4	P	44.8	3.6	570	570	209	209	2800	16	170	51.4	23.1
EBR-II-d8	2200	900	140	180	148	2ø8	2ø6	50 × 1.4	P	44.8	3.6	570	570	209	209	2800	16	170	41.9	18.9

Note: Some values have been rounded for a better presentation of the data. Detailed values can be found in the references. ^1^ CFRP type. P = Pre-cured, W = Wet lay-up. ^2^ Supposed values.

**Table 7:** Predicted values for ICD failure parameters in the studied beams according to the different approaches.

Specimens	S1				S2				MA1							MA2			
	*x_failure_*	*F_fR_*	*P_u,th_*	*P_u,exp_*	*x_failure_*	*F_fR_*	*P_u,th_*	*P_u,exp_*	*x_failure_*	∆*F_fR_*	∆*F_f,B_*	∆*F_f,F_*	∆*F_f,C_*	*P_u,th_*	*P_u,exp_*	*x_failure_*	∆*F_fR_*	*P_u,th_*	*P_u,exp_*
	(mm)	(kN)	(kN)	/*P_u,th_*	(mm)	(kN)	(kN)	/*P_u,th_*	(mm)	(kN)	(kN)	(kN)	(kN)	(kN)	/*P_u,th_*	(mm)	(kN)	(kN)	/*P_u,th_*
Al-Saawani et al. (2015) [58]																			
S-0.5-35-240	1000	253.0	200.3	1.1	1000	280.9	213.0	1.0	823	93.8	39.2	20.1	34.5	247.0	0.9	1000	61.9	183.4	1.2
S-0.9-35-240	1000	253.0	252.6	1.0	1000	280.9	265.2	1.0	1000	64.4	29.3	10.4	24.7	272.4	1.0	1000	44.5	230.2	1.1
S-0.9-24-240	1000	208.4	225.8	1.1	1000	245.7	241.8	1.0	1000	54.2	23.4	10.1	20.7	245.0	1.0	1000	37.7	221.7	1.1
S-0.9-17-240	1000	177.5	206.7	1.2	1000	221.9	225.2	1.1	1000	42.8	19.8	6.3	16.7	221.9	1.1	1000	33.3	215.5	1.2
S-1.3-35-240	1000	253.0	303.1	1.0	1000	280.9	315.3	1.0	1000	52.6	31.4	4.5	16.7	309.5	1.0	1000	36.5	278.5	1.1
SN-0.9-35-240	1640	253.0	253.6	1.0	1640	280.9	265.9	1.0	1537	53.5	19.1	12.8	21.6	347.8	0.7	1640	39.1	271.3	1.0
Al-Zaid et al. (2014) [59]																			
B-0.6-0	2000	468.6	179.7	1.4	2000	532.2	192.8	1.3	1720	156.2	48.0	38.8	69.5	234.6	1.1	2000	102.0	234.6	1.1
B-0.3-0	2000	234.3	132.2	1.3	2000	269.7	140.1	1.2	1580	77.8	23.5	19.6	34.7	189.5	0.9	2000	51.0	156.1	1.1
Aram et al. (2008) [50]																			
B3	667	64.5	51.8	1.2	667	84.3	60.3	1.0	667	20.8	10.5	2.0	8.4	50.2	1.3	667	13.8	45.4	1.4
B4	667	56.4	49.1	1.2	667	73.2	56.5	1.0	667	21.9	8.9	2.2	10.8	49.8	1.2	667	14.1	42.4	1.4
Bilotta et al. (2015) [53]																			
EBR_c_1.4×40_1	925	29.5	19.2	1.9	925	41.6	25.7	1.4	925	7.3	2.1	0.9	4.3	29.7	1.2	925	5.3	29.7	1.2
El-Zeadani (2019) [54]																			
CFRP-B1	1750	44.0	68.1	1.2	1750	55.3	71.7	1.2	1750	21.4	4.9	7.6	8.9	74.5	1.1	1750	13.4	71.4	1.2
Fu et al. (2017) [56]																			
B1S1	1300	189.3	301.2	0.9	1300	162.0	283.4	1.0	1300	44.9	24.9	7.1	13.0	305.4	0.9	1300	37.5	264.2	1.0
B1S2	1300	140.6	204.7	1.2	1300	131.0	198.6	1.2	1300	50.9	13.5	18.2	19.2	248.4	1.0	1300	34.9	185.5	1.3
Fu et al. (2018) [55]																			
LP2SP1750	1750	175.5	157.5	1.0	1750	151.0	145.5	1.0	1750	41.8	16.9	9.7	15.3	189.0	0.8	1750	35.8	163.7	0.9
LP2SP1250	1250	177.5	221.7	1.0	1250	152.6	204.7	1.1	1250	46.2	27.2	8.5	10.5	212.6	1.0	1250	36.2	176.9	1.2
LP2SP1000	1000	175.6	276.0	1.0	1000	150.6	254.6	1.1	1000	51.3	35.6	7.2	8.5	238.4	1.2	1000	36.0	213.4	1.3
LP4SP1000	1500	176.3	276.6	1.1	1500	151.1	255.0	1.2	1500	42.1	13.7	9.8	18.6	374.1	0.8	1500	36.1	325.9	0.9
LP8SP1000	1750	176.8	277.0	1.1	1750	151.5	255.4	1.2	876	47.2	29.3	8.0	9.8	373.6	0.8	876	36.2	315.1	1.0
Hong (2012) [45]																			
BPS60	1500	44.0	52.7	1.2	1500	58.3	58.2	1.1	1500	17.5	4.6	6.7	6.2	60.1	1.1	1500	11.8	52.2	1.2
BPS90	1500	44.0	52.7	1.1	1500	58.3	58.2	1.0	1500	17.5	4.6	6.7	6.2	60.1	1.0	1500	11.8	52.2	1.2
BPD90	1500	62.3	57.7	1.3	1500	82.5	66.2	1.1	1500	16.1	6.7	5.3	4.1	64.5	1.1	1500	11.8	62.0	1.2
BPDW90	1500	88.0	68.2	1.4	1500	98.6	72.1	1.3	1004	34.7	10.4	12.6	11.6	116.0	0.8	1500	23.6	78.7	1.2
Kotynia et al. (2009) [52]																			
B-08S	1400	49.1	77.0	1.2	1400	60.1	81.5	1.2	1400	12.1	7.5	1.5	3.1	75.2	1.3	1400	8.4	72.3	1.3
B-08M	1400	157.7	117.2	1.2	1400	165.2	120.0	1.2	1400	33.0	12.8	6.3	13.8	150.2	0.9	1400	23.6	115.3	1.2
Maalej et al. (2005) [44]																			
A3	500	41.6	74.3	1.0	500	41.6	74.3	1.0	500	32.4	13.8	1.5	17.1	74.3	1.0	500	20.8	74.3	1.0
A4	500	41.6	74.3	1.0	500	41.6	74.3	1.0	500	32.4	13.8	1.5	17.1	74.3	1.0	500	20.8	74.3	1.0
A5	500	69.7	87.2	1.0	500	69.7	87.2	1.0	500	31.8	15.5	1.2	15.1	87.2	1.0	500	20.8	87.2	1.0
A6	500	69.7	87.2	1.0	500	69.7	87.2	1.0	500	31.8	15.5	1.2	15.1	87.2	1.0	500	20.8	87.2	1.0
B3	1000	168.6	296.9	0.9	1000	152.2	296.9	0.9	1000	68.5	28.6	8.8	31.1	296.9	0.9	1000	47.0	271.2	1.0
B4	1000	168.6	296.9	0.9	1000	152.2	296.9	0.9	1000	68.5	28.6	8.8	31.1	296.9	0.9	1000	47.0	271.2	1.0
B5	1000	253.0	335.1	0.9	1000	215.2	335.1	0.9	1000	67.3	32.6	8.1	26.6	345.1	0.9	1000	47.0	299.3	1.0
B6	1000	253.0	335.1	0.8	1000	215.2	335.1	0.8	1000	67.3	32.6	8.1	26.6	345.1	0.8	1000	47.0	299.3	1.0
C3	1600	372.0	740.1	0.9	1600	321.3	740.1	0.9	1600	111.4	53.9	20.1	37.4	741.8	0.9	1600	85.6	683.8	1.0
C4	1600	372.0	740.1	0.9	1600	321.3	740.1	0.9	1600	111.4	53.9	20.1	37.4	741.8	0.9	1600	85.6	683.8	1.0
C5	1600	526.0	808.8	0.8	1600	454.4	808.8	0.8	1600	118.4	75.6	16.7	26.1	794.1	0.8	1600	85.6	760.8	0.9
Niu et al. (2006) [51]																			
A1	2100	202.1	94.0	1.4	2100	277.4	107.8	1.2	1330	119.1	27.0	32.2	59.8	167.8	0.8	2100	69.9	105.3	1.2
A2	2100	207.6	95.3	1.4	2100	282.9	109.1	1.2	1320	115.4	32.0	30.1	53.4	160.4	0.8	2100	70.8	105.8	1.2
A3	2100	269.7	107.9	1.0	2100	272.7	108.5	0.9	1837	200.4	49.5	47.6	103.2	112.2	0.9	2100	124.1	112.2	0.9
A4	2100	426.0	133.2	1.0	2100	432.1	134.3	1.0	1577	190.3	64.2	44.9	81.2	173.2	0.8	2100	123.1	170.0	0.8
A5	2100	289.7	110.3	1.0	2100	313.8	114.7	0.9	1836	132.6	35.8	30.9	65.9	137.0	0.8	2100	83.4	121.0	0.9
A6	2100	179.6	92.1	1.0	2100	194.7	0.0	1.0	1837	132.6	39.9	30.3	62.3	95.0	1.0	2100	82.7	95.0	1.0
B1	1600	212.6	126.6	1.1	1600	287.6	144.7	1.0	1075	119.9	34.7	28.8	56.4	202.0	0.7	1600	71.7	124.4	1.2
B2	1600	267.2	140.9	0.8	1600	270.9	141.8	0.8	1338	200.1	62.4	45.2	92.5	147.1	0.8	1600	123.3	147.1	0.8
C2	2100	207.4	101.1	1.3	2100	282.6	114.9	1.2	1286	77.5	27.3	16.6	33.7	174.2	0.8	2100	50.9	119.6	1.1
C3	2100	265.8	113.0	0.9	2100	269.8	113.7	0.9	1773	132.3	47.7	26.6	58.0	117.9	0.9	2100	89.6	117.9	0.9
C4	2100	178.1	97.7	0.9	2100	193.9	100.7	0.9	1936	92.1	28.5	18.4	45.2	101.0	0.9	2100	60.1	101.0	0.9
Oller (2005) [43]																			
1D2	1000	100.8	93.7	1.2	1000	124.7	102.5	1.1	1000	38.2	14.1	6.8	17.3	98.3	1.1	1000	24.3	90.0	1.2
1C1	1000	100.8	93.7	1.1	1000	124.7	102.5	1.0	1000	38.2	14.1	6.8	17.3	98.3	1.1	1000	24.3	90.0	1.2
1B1	1000	100.8	93.7	1.1	1000	124.7	102.5	1.0	1000	38.2	14.1	6.8	17.3	98.3	1.0	1000	24.3	90.0	1.1
1A	1000	100.8	93.7	1.2	1000	124.7	102.5	1.1	1000	38.2	14.1	6.8	17.3	98.3	1.1	1000	24.3	90.0	1.2
2D1	1000	100.8	121.3	1.1	1000	124.7	129.7	1.0	1000	32.8	14.4	4.6	13.8	123.1	1.0	1000	21.1	117.6	1.1
2D2	1000	201.6	153.4	1.1	1000	223.0	160.3	1.0	883	67.4	26.8	9.7	30.8	181.6	0.9	1000	42.3	149.3	1.1
2C1	1000	100.8	121.3	1.2	1000	124.7	129.7	1.1	1000	32.8	14.4	4.6	13.8	123.1	1.2	1000	21.1	117.6	1.2
Peng et al. (2014) [47]																			
PRS-EB	1300	38.1	100.4	1.5	1300	51.2	107.1	1.4	1300	9.5	5.2	1.9	2.4	100.7	1.5	1300	7.2	99.3	1.5
Rusinowski and Täljsten (2009) [49]																			
Beam 2	950	124.5	65.7	1.1	950	132.2	67.9	1.1	867	31.6	12.2	2.5	16.9	84.3	0.9	950	21.6	73.2	1.0
Beam 6	950	124.5	65.7	1.1	950	132.2	67.9	1.0	867	31.6	12.2	2.5	16.9	84.3	0.8	950	21.6	73.2	1.0
Slaitas and Valivonis (2020) [57]																			
BS-0	955	58.1	123.5	1.0	955	71.7	131.9	0.9	955	17.5	10.4	2.4	4.7	117.9	1.0	955	12.3	114.0	1.1
Turco et al. (2017) [48]																			
BL_A-w	762	43.1	98.0	1.1	762	42.0	97.2	1.2	762	20.3	5.7	4.6	10.1	106.6	1.0	762	12.5	85.5	1.3
BL_2A-w	762	60.9	109.8	1.0	762	59.4	108.7	1.0	762	18.9	11.3	2.7	4.9	102.1	1.0	762	12.5	94.3	1.1
BH_2A-w	762	75.0	123.1	1.0	762	68.8	118.4	1.0	762	22.8	13.8	2.7	6.3	113.2	1.0	762	15.9	99.1	1.2
SL_A-w	762	43.1	48.9	1.0	762	49.5	51.4	1.0	620	37.3	9.3	5.3	22.7	62.2	0.8	762	18.0	44.9	1.1
SL_2A-2w	762	85.5	64.0	0.9	762	86.9	64.5	0.9	620	72.1	14.3	11.9	45.9	81.7	0.7	762	35.7	69.2	0.8
SL_2A-w	762	60.9	54.9	1.0	762	69.9	58.3	1.0	620	31.7	11.6	4.5	15.6	68.0	0.8	762	18.0	49.2	1.1
Zhou et al. (2020) [46]																			
B2	1200	48.4	108.8	1.0	1200	47.5	108.4	1.0	1200	50.7	9.1	11.4	30.2	112.0	0.9	1200	25.7	104.6	1.0
Present study																			
EBR-I-d10	900	47.3	38.0	1.2	900	57.4	42.5	1.1	900	17.3	4.9	3.5	8.9	45.6	1.0	900	10.5	42.1	1.1
EBR-II-d10	900	60.9	48.5	1.1	900	70.7	52.1	1.0	900	17.3	7.5	2.0	7.8	51.6	1.0	900	11.7	45.4	1.1
EBR-II-d8	900	60.9	39.7	1.1	900	70.7	43.4	1.0	761	20.0	8.7	2.6	8.7	48.4	0.9	900	13.3	36.9	1.1

**Table 8:** Statistics of experimental vs. theoretical results for all specimens in the database.

			S1	S2	MA1	MA2
^1^	Specimens	(-)	68	68	68	68
	Mean value	(-)	1.09	1.04	0.96	1.09
	StDev	(-)	0.18	0.13	0.15	0.15
	CoV	(-)	0.17	0.12	0.16	0.14
^2^	Specimens	(-)	62 (91%)	63 (93%)	43 (63%)	57 (84%)
	Mean value	(-)	1.10	1.05	1.00	1.11
	StDev	(-)	0.18	0.13	0.15	0.15
	CoV	(-)	0.16	0.12	0.15	0.14

^1^ These values have been calculated considering the theoretical failure load as the minimum of ICD or concrete crushing (CC)/fibre rupture (FR). ^2^ These values have been calculated considering only the beams which theoretically failed by ICD.

## Data Availability

The raw/processed data required to reproduce these findings cannot be shared at this time as the data also form part of an ongoing study.
